# A computational modeling framework for pre-clinical evaluation of cardiac mapping systems

**DOI:** 10.3389/fphys.2023.1074527

**Published:** 2023-07-06

**Authors:** Suran Galappaththige, Pras Pathmanathan, Richard A. Gray

**Affiliations:** Division of Biomedical Physics, Office of Science and Engineering Laboratories, Center for Devices and Radiological Health, Food and Drug Administration, Silver Spring, MD, United States

**Keywords:** cardiac mapping, computational modeling, cardiac electrophysiology, arrhythmia, regulatory science

## Abstract

There are a variety of difficulties in evaluating clinical cardiac mapping systems, most notably the inability to record the transmembrane potential throughout the entire heart during patient procedures which prevents the comparison to a relevant “gold standard”. Cardiac mapping systems are comprised of hardware and software elements including sophisticated mathematical algorithms, both of which continue to undergo rapid innovation. The purpose of this study is to develop a computational modeling framework to evaluate the performance of cardiac mapping systems. The framework enables rigorous evaluation of a mapping system’s ability to localize and characterize (i.e., focal or reentrant) arrhythmogenic sources in the heart. The main component of our tool is a library of computer simulations of various dynamic patterns throughout the entire heart in which the type and location of the arrhythmogenic sources are known. Our framework allows for performance evaluation for various electrode configurations, heart geometries, arrhythmias, and electrogram noise levels and involves blind comparison of mapping systems against a “silver standard” comprised of computer simulations in which the precise transmembrane potential patterns throughout the heart are known. A feasibility study was performed using simulations of patterns in the human left atria and three hypothetical virtual catheter electrode arrays. Activation times (AcT) and patterns (AcP) were computed for three virtual electrode arrays: two basket arrays with good and poor contact and one high-resolution grid with uniform spacing. The average root mean squared difference of AcTs of electrograms and those of the nearest endocardial action potential was less than 1 ms and therefore appears to be a poor performance metric. In an effort to standardize performance evaluation of mapping systems a novel performance metric is introduced based on the number of AcPs identified correctly and those considered spurious as well as misclassifications of arrhythmia type; spatial and temporal localization accuracy of correctly identified patterns was also quantified. This approach provides a rigorous quantitative analysis of cardiac mapping system performance. Proof of concept of this computational evaluation framework suggests that it could help safeguard that mapping systems perform as expected as well as provide estimates of system accuracy.

## 1 Introduction

Catheter ablation is a primary therapy for the treatment of cardiac arrhythmias. Life threatening ventricular tachycardia and fibrillation occur for a variety of reasons including heart failure and affect millions of individuals each year. The most common arrhythmia is atrial fibrillation (AF) with an estimated prevalence in the United States (U.S) alone of 3–5 million (Calkins et al., 2017), and the deadliest arrhythmia is ventricular fibrillation (VF) which is the leading cause of death in the U.S. There has been a substantial increase in the annual number of in-hospital catheter ablation procedures (Kneeland and Fang, 2009; Deshmukh et al., 2013; Hosseini et al., 2017; Breithardt and Borggrefe, 2021) and experimental data and ablation outcomes suggest that multi-electrode cardiac mapping systems, that provide simultaneous acquisition of tens or hundreds of recording sites, is responsible for this increase (Calkins et al., 2017; Rolf et al., 2019). Studies have shown that electroanatomical mapping systems significantly reduce procedure duration and radiation exposure compared to conventional fluoroscopy-guided atrial fibrillation (AF) ablation procedures (Rotter et al., 2005; Estner et al., 2006). Cardiac mapping is necessary to locate the sources of arrhythmias for ablation and multipolar catheters, such as those incorporated into electroanatomical mapping systems, allow rapid identification of complex spatial patterns of electrical activity and structural abnormalities (e.g., scar tissue) during fibrillation. Purported mechanisms of electrical impulse propagation during arrhythmias include (Schotten et al., 2021): 1) stable reentrant waves (either anatomical or functional) sometimes accompanied by fibrillatory conduction; 2) unstable reentry; 3) single or multiple foci with or without fibrillatory conduction; and 4) asynchronous activation of the endocardium and epicardium due to transmural electrical dissociation.

There is considerable debate regarding the underlying activation patterns of clinical AF and there are inconsistencies in ablation outcomes in different studies (Roney et al., 2020). A variety of factors are thought to underly these uncertainties including catheter electrode density (Barbhayia et al., 2015; Roney et al., 2017a; Aronis et al., 2019) and significant differences in mapping system catheters and algorithms, most notably phase mapping. For example, studies directly comparing two mapping algorithms using the same raw data from catheter electrodes in clinical studies indicate variability in concordance/discordance at both ablation sites and elsewhere (Alhusseini et al., 2017; Bellmann et al., 2018; Swerdlow et al., 2019). In another example, Martinez-Mateu et al. demonstrated in computational modeling studies that “far-field contributions to electrograms during AF reduce the accuracy of detecting and interpreting reentrant activity.” (Martinez-Mateu et al., 2019) The early success of phase mapping during clinical VF (Masse et al., 2007) has not been replicated for clinical AF, probably for a variety of interrelated reasons including: the differences in ventricular and atrial geometry; possible differences in underlying mechanisms; and differences in electrogram signal characteristics (Gray et al., 1998; Umapathy et al., 2010). Numerous authors have discussed further difficulties of implementing phase mapping during clinical AF (Roney et al., 2017b; Jacquemet, 2018; Podziemski et al., 2018; Li et al., 2020; Roney et al., 2020) and Child et al. conclude “Despite phase analysis being the preferred method in mapping AF, there are significant challenges in this approach because of the non-sinusoidal and fractionated nature of the recorded signal. Several complex signal transformations and analytical methods have been used in response to these difficulties reporting conflicting results, and there is urgent need to validate and standardize these techniques.” (Child et al., 2018).

**FIGURE 1:**
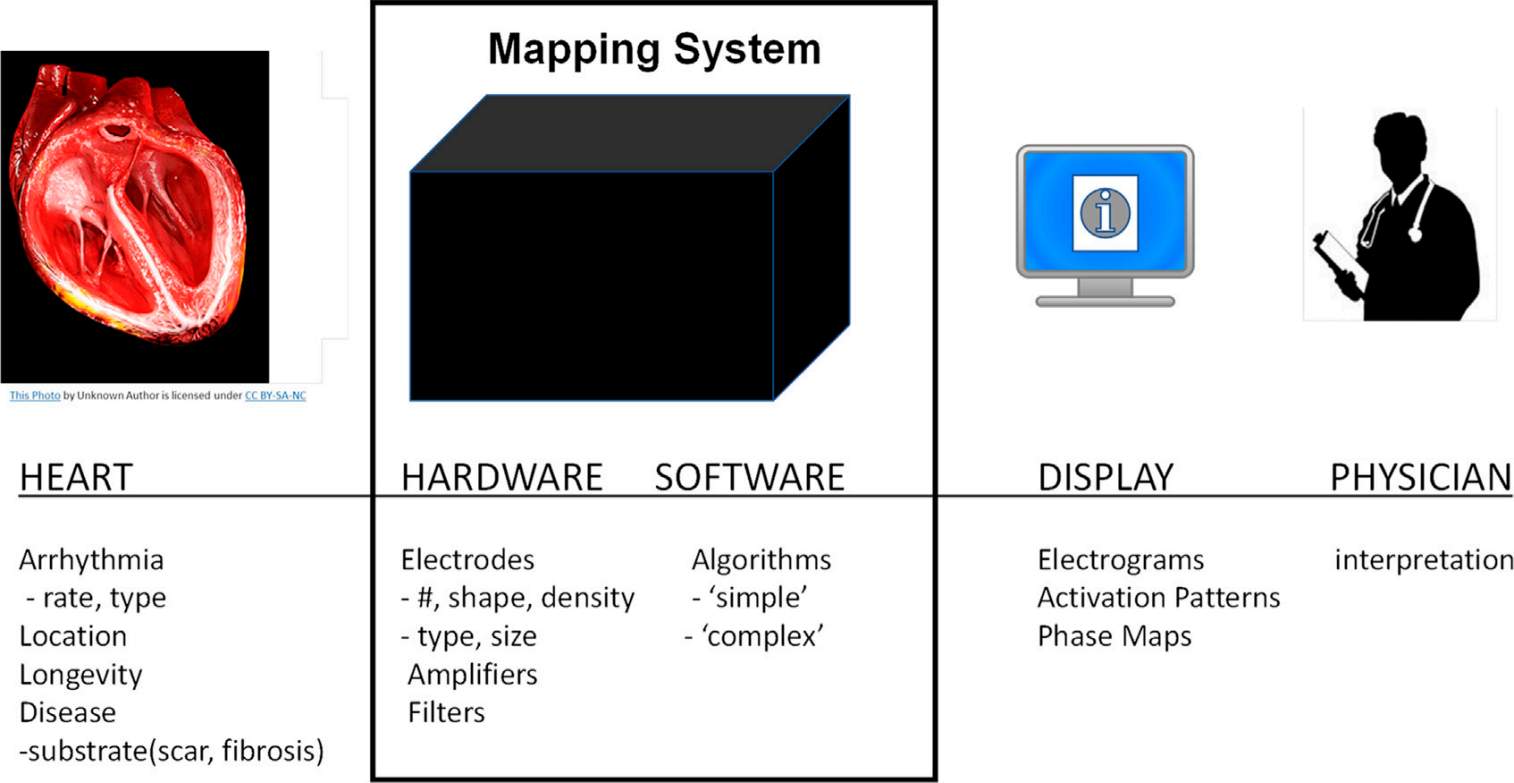
Overview of cardiac mapping system use. Cardiac mapping systems include both hardware and software elements are used to record electrograms from the patient’s heart and display a variety of information to the cardiac electrophysiological physician.

The performance of cardiac mapping systems depends on numerous complex and inter-related factors including the patient’s condition, the mapping system hardware and software including numerous mathematical algorithms, and the interpretation of the mapping system output by the physician (see Figure 1). Typically, performance analysis of a new mapping system involves interpretation of system output by multiple electrophysiological physicians. The ability to quantitatively evaluate the performance of mapping systems in the intended population is challenging, if not impossible, however, a computational framework that can quantitatively integrate these multifactorial complexities has the potential to provide concrete performance metrics for cardiac mapping systems. Here we present a novel computational modeling framework that enables quantitative assessment of the accuracy of cardiac mapping systems and demonstrate a “proof-of-concept” using a hypothetical example. Our proposed framework allows for blinded system evaluation and is based on estimating mapping algorithm performance using simulated electrograms derived from computer simulations in which the precise transmembrane potential patterns are known.

## 2 Methods

### 2.1 Overview of proposed Mapping System Evaluation Framework (MSEF)

Here we present a Mapping System Evaluation Framework (MSEF) to quantitatively evaluate clinical cardiac mapping systems using computational models. Our proposed framework includes the ability to evaluate mapping system performance under: 1) various electrode configurations; 2) various heart geometries; 3) various arrhythmias; and 4) the effect of noise on system performance. MSEF allows for blind testing of cardiac mapping system performance against a ‘silver standard’ in which the transmembrane potential is known throughout the entire heart. Our framework takes advantage of the fact that the methodology for quantifying the dynamic spatial patterns of transmembrane potential throughout the heart are well-established and robust, as exemplified in hundreds of experimental (e.g., optical mapping) and numerical (e.g., computational modeling) studies.

The framework includes a “library” of pre-computed simulations incorporating a range of activation patterns including paced beats, reentry, and “focal” beats replicated via pacing. For each simulation, activation times for each node in the computational mesh are computed using the maximum upstroke velocity of each action potential. The location of reentrant beats are computed via the computation of phase maps, identifying surface phase singularities, and then computing their “center of mass” from phase singularity density maps. Each entry in the library consists of: 1) transmembrane potential at every node sampled at 1 kHz; 2) activation times at every node; 3) the location of all paced beats (including simulated focal activity); and 4) the surface location and chirality of all reentrant waves.

Table 1 provides the chronological list of steps in the overall process of evaluating a generic cardiac mapping system (MS) using the MSEF. The process includes two participants: the “User” which is most likely the MS developer and; 2) the MSEF “Administrator”.

**TABLE 1:** Steps for MSEF execution in chronological order.

1) The User identifies the following information relevant for their mapping system (MS)
a. The heart chamber(s)
b. Type of activity (e.g., sinus rhythm, pacing, atrial tachycardia, etc.)
c. Recording electrode type(s) (e.g., contact endocardial)
2) The Administrator selects a number of simulations from the library based on the information contained in 1)
3) The Administrator provides the User with the set of points representing the heart surface(s) corresponding to the simulations selected in 2)
4) The User identifies the location(s) of the electrode(s) in their MS in the same three-dimensional space as the data in 3) so that the relative electrode location(s) and heart chamber geometry are known. For example, the User could ‘align’ their MS electrodes to the 3-D heart geometry digitally using visualization software with a CAD representation of their electrode catheter or physically using a 3-D printed version of the heart geometry and their actual catheters. The User provides these locations to the Tool Administrator. In the case of ‘roving’ catheters this information will include locations as a function of time
5) The User characterizes the noise level for each of electrode locations, which may vary across locations, and also provides these noise levels to the Administrator
6) For EACH simulation selected in 2), and based on the information contained in 1) and 4), the Administrator computes the virtual electrograms corresponding to the location(s) provided by the User in 4) and sends these electrogram(s) to the User such that the User is blind to the specifics of the underlying electrical activity in the simulation. ‘Virtual noise’ is added to each electrogram based on the information provided by the User in 5)
7) The User processes the electrograms sent by the Administrator in 6) either by using a digital to analog converter and inputting these signals into their physical MS or via inputting them directly into their software. In either case the User will bypass the physical electrode(s) in their system
8) The User sends the following system output to the Administrator
a. Predicted activation times at specific locations on the heart surface (i.e., a subset of points in 3) corresponding to their MS. For example, for non-contact electrodes these locations will be different than the electrode locations provided in 4)
b. Predicted type(s) of electrical activation patterns and their location(s) as a function of time in relation to the surface points that were provided in 3)
9) The Administrator runs a set of ‘comparison’ tools which include
a. Computing the root mean square error (RMSE) in activation times computed for all points provided in 8.a) as well as the average RMSE per electrode and number of ‘missed’ activations and spurious (i.e., wrong) activations by comparing the activation times computed from the virtual transmembrane potential from the same sites
b. Comparing the type(s), location(s), and timing of electrical activation patterns provided by the User to those computed from the corresponding computer simulations, as well as identifying missed and spurious patterns as well as those that were misclassified
c. Computing the spatial and temporal distances between the source(s) of activity patterns correctly predicted by the User

### 2.2 Pilot study to demonstrate the feasibility of MSEF

To demonstrate the feasibility of this framework, we present a specific implementation of the approach described above in this manuscript. Due to the large number of variables identified in Step 1) above, a comprehensive assessment of MSEF is beyond the scope of this study. The implementation presented here is comprised of: 1) two simulations of electrical activity in a healthy isotropic human left atria (2 seconds duration) comprised of paced (P), reentrant (R), and focal (F) beats; 2) three virtual electrode catheters: two idealized 64 basket arrays and one high-resolution 6 × 6 array; 3) well established algorithms to compute activation times and localize reentry from high resolution transmembrane patterns; and 4) simple generic mapping system algorithms. Video movies of these two simulations are provided in the Supplementary Material.

### 2.3 Simulations

The monodomain equation governing electrical activation and propagation in excitable tissue was solved:

$$\chi \left(C_{m}\;\frac{\partial V}{\partial t}+I_{ion}+I_{stim}\right)-\nabla .\;\left(\sigma \nabla V_{m}\right)=0$$ (1)

where *V_m_* is the transmembrane voltage, χ = 1,400 cm^−1^ is the surface-area-to-volume ratio, and C_*m*_ = 1.0 μF cm^−2^ is the capacitance per unit area. *I*_*ion*_ is the ionic current computed by coupling the monodomain equation with the Nygren cell model (Nygren et al., 1998) of an adult human atrial cell; *I*_*stim*_ is the stimulus current imposed during S1 and S2 stimulation. The conductivity was chosen to be isotropic with a value of 0.466 mS cm^−1^ to match the conduction velocity of human atria of 55 cm/s (McDowell et al., 2015). The monodomain equation was solved using the finite element method using the Chaste software package (Mirams et al., 2013). Simulations were run on a high-resolution computational mesh of a human left atrium derived from a commercially available computer aided design (CAD) model by Zygote Cooperation. The CAD model was imported into Tetgen (Si, 2015) and an unstructured tetrahedral mesh consisting of 1.32 million nodes and 4.6 million elements with an average edge length of 252 µm was generated. The thickness was nonuniform and derived from patient specific imaging. The partial differential equations were solved using a backward Euler discretization with timesteps of 0.1 ms for both the partial differential equations and the cell model. The transmembrane voltage of each node was saved every 1 ms and simulations were run for a total of 2 s.

Two simulations were performed and videos of these are provided in the Supplementary Material. The first simulation is comprised of a single paced beat followed by 6 beats of figure-of-eight reentry, i.e., a pair of counter rotating reentrant waves (one clockwise denoted as “R+” and one counterclockwise denoted as “R-” when viewed from the endocardium), generated via an S1-S2 stimulus protocol. The paced beat was initiated at the junction of the posterior left atrium and the left inferior pulmonary vein and is referred to as “P” and the S2 was applied in the free wall of the septum of the left atria (LA). The second simulation is simulated focal activity and was constructed to allow a direct comparison with the reentrant beats. We simultaneously paced the locations corresponding to the center of mass of R+ and R-with inter beat intervals corresponding to the reentrant cycle lengths of each of the six reentrant beats; we refer to these patterns as “F+” and “F-”. Overall we simulated 13 activation patterns (AcPs) across the two simulations: one paced beat (P), six figure-of-eight patterns with clockwise (R+) and counterclockwise (R-) activation patterns and 6 pairs of focal beats (F+ and F-).

### 2.4 Electrode configurations and electrograms

**FIGURE 2:**
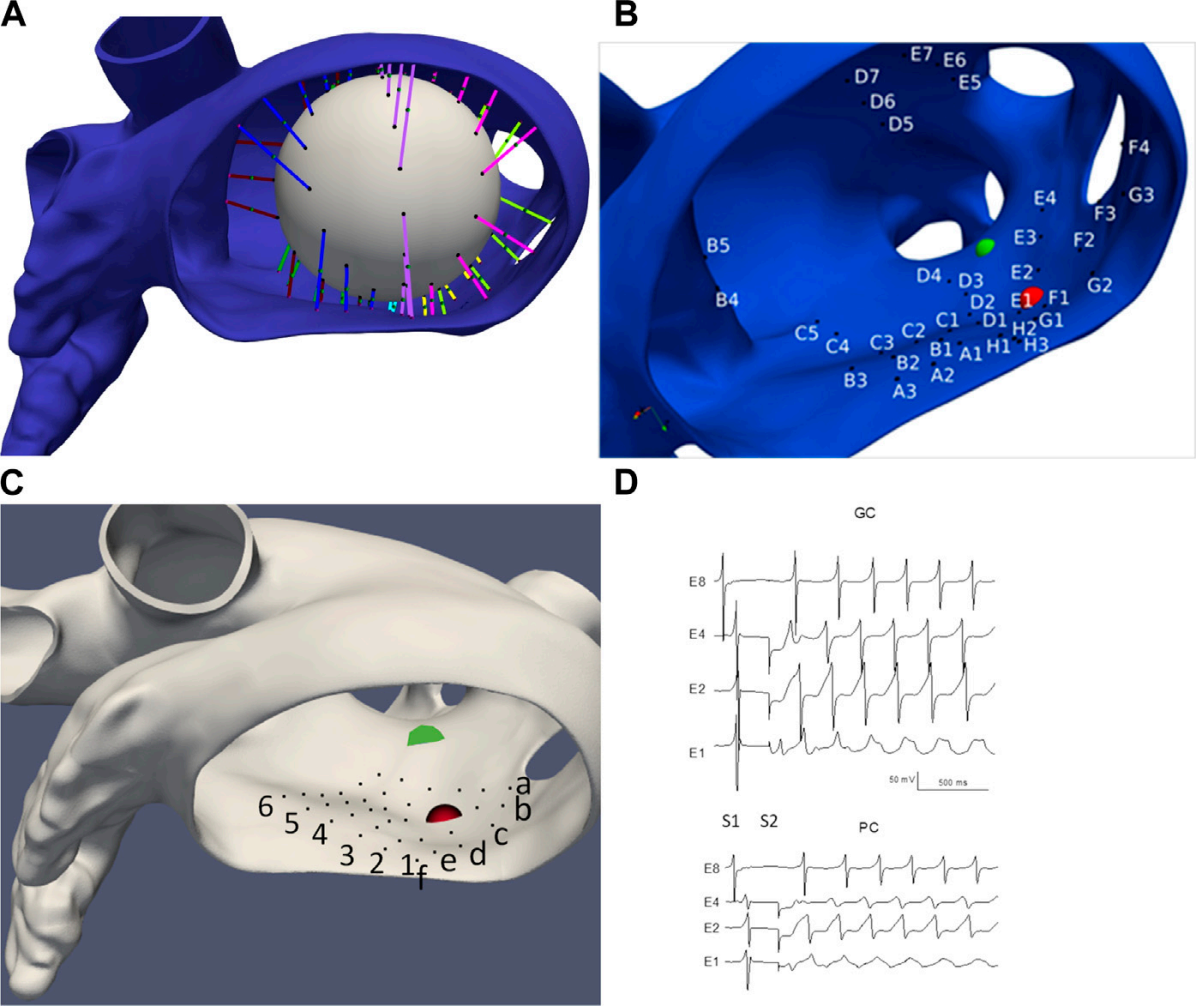
LA geometry and virtual electrode locations. **(A)** LA with the projection lines (colored according to spline #) from the sphere to the endocardial surface. **(B)** Location of BGC electrode locations (A1-H8). **(C)** Location of UA electrode locations (a1-f6). Endocardial site R+ is shown as a red sphere and R-as a green sphere. **(D)** Examples of electrograms from basket electrodes: Good Contact (BGC) and Poor Contact (BPC) with noise added; noise level was 1 mV/ms for BGC and 0.5 mV/ms for BPC.

We choose two idealized generic basket electrode geometries comprised of 64 unipolar electrodes (8 electrodes spaced 2 mm apart on 8 separate splines) with a diameter 38 mm and one idealized “grid” electrode geometry comprised of 36 electrodes aligned in a 6 × 6 grid 4 mm apart. Basket catheters expand within the heart chamber into which they are placed and the distance between each electrode and the heart surface varies depending on the electrode spacing and the endocardial geometry. We initially considered the “worst-case” as all 64 electrodes residing on a 38 mm sphere “centered” in the LA; however, this case provided meaningless results which are not presented here. We consider the “best case” by finding the 64 LA sites on the mesh that minimize the distance from the endocardial surface to each electrode (see Figure 2), and then placing the electrodes 0.5 mm from the heart surface; we refer to this case as “basket good contact” (BGC). We also consider an “intermediate” case by placing the 64 locations at the midpoint of the line connecting the point on the sphere to the nearest endocardial site (see colored lines in Figure 2A); we refer to this case as “basket poor contact” (BPC). Although a sphere was used to derive the locations for BGC and BPC, the resulting electrode locations do not lie on a sphere; as such the distance between electrodes on a spline are not constant. The 8 × 8 electrode arrays for BGC and BPC are represented in 2-D arrays labeled A1 to H8 (see Figure 2B). Finally, we studied an idealized localized electrode ‘uniform array’ (referred to as “UA”) in which a 2 cm × 2 cm square was manually placed close to the endocardial surface over R+ and then each of the 36 virtual electrodes were moved such that they were 0.5 mm from the nearest endocardial site. The 6 × 6 electrode array for UA is represented in a 2-D array labeled a1 to f6 and shown in Figure 2C. Examples of electrograms from BGC and BPC with noise added are shown in Figure 2D.

Virtual electrograms (referred to as electrograms in this manuscript) were computed for all electrode locations for the three catheter configurations for the two simulations as:

$$\emptyset_{e}\left(x^{\prime },y^{\prime },z^{\prime }\right)=\int \left(-\sigma_{e}\nabla V_{m}\right).\left[\nabla \frac{1}{r}\right]dx\;dy\;dz$$ (2)

where

$$r={\left[{\left({x-x}^{\prime }\right)}^{2}+{\left({y-y}^{\prime }\right)}^{2}+{\left({z-z}^{\prime }\right)}^{2}\right]}^{1/2}$$ (3)

where $\emptyset_{e}$ is the extracellular unipolar potential (i.e., electrogram), $\nabla V_{m}$ is the spatial gradient of $V_{m}$ , $\sigma_{e}=$ 7 m/cm is the extracellular conductivity, *r* is the distance from a “source” point $\left(x,y,z\right)$ within the heart to the electrode location, $\left(x^{\prime },y^{\prime },z^{\prime }\right)$ , and the integral is over the myocardium. This computation ignores the size of the electrode assuming it is a point. The integral was computed by summing the volume integrals over each element in the finite element mesh, calculated using Gaussian quadrature and using the finite element solution for $V_{m}$ (linear in each element).

### 2.5 Algorithms

The value of activation times (AcTs) for $V_{m}$ were computed as the time of maximum derivative of $V_{m}$ provided it was greater than a threshold value (α) with the constraint that two activations did not occur within a specific interval (β). The values of α and β were selected based on a sensitivity analysis performed on seven $V_{m}$ sites from the first simulation at five locations within the reentrant isthmus and two sites outside the isthmus. Specifically, we computed AcTs for thresholds of α = 0.1, 0.2, 0.3, and 0.5 mV/ms and for β = of 25, 50, 100, 150, 200, 250, and 500 ms. For all seven sites, the number and values of AcTs were the same for thresholds of 0.1, 0.2, and 0.3 mV/ms and window sizes of 100 and 150 m. Therefore, we choose values of α = 0.2 mV/ms and β = 100 m for the computation of AcTs from $V_{m}$ signals (Dube et al., 2009). Interpolation between samples was not employed so the resolution of AcTs was 1 ms.

The algorithm for identifying reentrant patterns for the simulations involved computing the 3-D filaments using state-space phase analysis using Eqn (Hosseini et al., 2017). as described previously (Pathmanathan and Gray, 2015; Galappaththige et al., 2019).

$$\theta \left(t\right)=\mathrm{atan}2\left[V_{m}\left(t+2\right)+30,V_{m}\left(t-2\right)+30\right]$$ (4)

where *θ* is a computed phase variable; endocardial phase singularities (PSs) were computed from the proper end of these filaments. PS density maps were computed using a custom Python script that calculated the number of times a PS occurred at each node within the simulation interval between 1 and 2 s. Since we identified a relatively stable figure-of-eight reentrant pattern via visual inspection, a k-means clustering algorithm was implemented to identify two clusters corresponding to clockwise (+) and counterclockwise (−) reentry on the endocardial surface. The center of mass of these two clusters were considered the locations of the two endocardial surface PS locations, R+, and R-.

The algorithm for computing AcTs from the electrograms was identical to that used for $V_{m}$ with the exception that the sign of the ‘derivative threshold’ was opposite; the value of *ß* was 100 m and the value of *a* was −1 mV/ms for BGC and *a* = −0.5 mV/ms for BPC and UA. Due to the significant differences between the morphology of $V_{m}$ and $\emptyset_{e}$ signals, we did not employ phase analysis for the algorithms to identify activation patterns from the electrogram data. Instead we developed very simple algorithms to identify focal (F) and reentry (R) patterns using only AcTs. The algorithms include two parameters (a ‘difference threshold’, δ in ms; and an interval, γ in ms). We identified the presence of both F and R patterns at each electrode location using the value of AcT at that site and the AcTs of the eight surrounding electrode neighbors. A site was classified as F if all the AcT differences of the 8 neighbors and the central pixel were between -δ and *γ*+*δ*. A site was classified as R+ (R−) if there was a clockwise (counterclockwise) progression of AcT’s along the path of the 8 neighboring electrodes including a continuation of activation between beats; specifically, each of the differences along the path had to be between -δ and *γ*+*δ*. These pattern identification algorithms include the computation of eight differences and we chose *δ* = 2 and *γ* = 100.

### 2.6 Addition of noise

Noise was included by adding uniformly distributed random values to $\emptyset_{e}$ . We choose the level of noise to be equal to the threshold value (*a*) which varied with electrode configuration (see above) which is a level at or above clinical values (Unger et al., 2019) although the effect of noise (as a factor of threshold) is included in the Supplement. Recall that in the actual use of our proposed framework the user will provide information on the actual level of noise for their MS to the MSEF.

### 2.7 Performance analysis

Evaluation of AcTs was performed for the electrograms for both BGC and BPC MSs by comparing to the corresponding values computed from $V_{m}$ . The acceptable level of difference in AcTs between the MSs and simulation is unclear and may depend on the activation pattern, therefore we introduce a “tolerance” variable (*Tol*) and analyzed performance as a function of *Tol*. The ability of each MS to identify AcTs was computed by identifying: 1) correctly identified AcTs; and 2) spurious AcTs. In addition, the average RMS of all correctly identified ACTs was computed. An “AcT Performance Metric” (AcTPM) was computed to assess the ability of a MS to identify AcTs:

$$AcTPM=fC\;*\left(1-fS\right)\;*\;100$$ (5)

where fC and fS are the fraction of correct and spurious AcTs, respectively. A value of 100 indicates perfect performance. Specifically, fC is computed as the number of AcTs for simulated electrogram that are within *Tol* of a corresponding AcT computed from $V_{m}$ of the nearest endocardial site divided by the total number of $V_{m}$ AcTs from that site; and fS is computed as the number of AcTs for a simulated electrogram that are *not* within *Tol* of a corresponding AcT computed from $V_{m}$ of the nearest endocardial site divided by the total number of $V_{m}$ AcTs from that site (if fS is >1, then fS is set equal to 1).

Activation patterns (AcPs) were computed at each site using the AcTs from the 3 × 3 array neighborhood and analyzed similarly and were considered correct if they were localized within 100 ms and if the distance to the true (x,y,z) location in the simulations was less than 1 cm. We define the temporal localization error (E_T_) as the difference between the electrogram AcT at the centralized site and the corresponding stimulation time (i.e., S1, S3, S4, S5) and the spatial localization error (E_X_) is the Euclidean distance between the centralized electrode location and the site identified as R+ or R-from the $V_{m}$ simulations (as described above). An ‘AcP Performance Metric’ (AcPPM) was computed to assess the ability of a MS to identify AcPs:

$$AcPPM=fC\;*\left(1-fS\right)\;*\left(1-fM\right)\;*\;100$$ (6)

where fC and fS are the fraction of correct and spurious AcPs and fM is the fraction of “misclassifications” defined as a wrong pattern type for a beat (matching the identification criteria for the above temporal and spatial distances of a different pattern). Specifically, a correct AcP from a virtual electrode array was defined as the identification of the identical pattern for the same beat for the “ground truth” (25 patterns: 1P, 6 R+, 6 R-, 6 F+, and 6 F-); and a spurious AcP from a virtual electrode array was defined as the identification of a pattern that did not correspond to the ground truth. AcTs and AcPs for BGC and BPC were compared to those computed for the simulation results for pacing, focal and reentrant patterns separately. In addition, the average temporal (E_T_) and spatial (E_X_) localization errors of the correctly identified and spurious AcPs were computed. Since the definition of the correct identification of a pattern depends on a 1 cm ‘tolerance’ the value of E_X_ is constrained (E_X_ ≤ 1).

## 3 Results

### 3.1 High-resolution simulations

**FIGURE 3:**
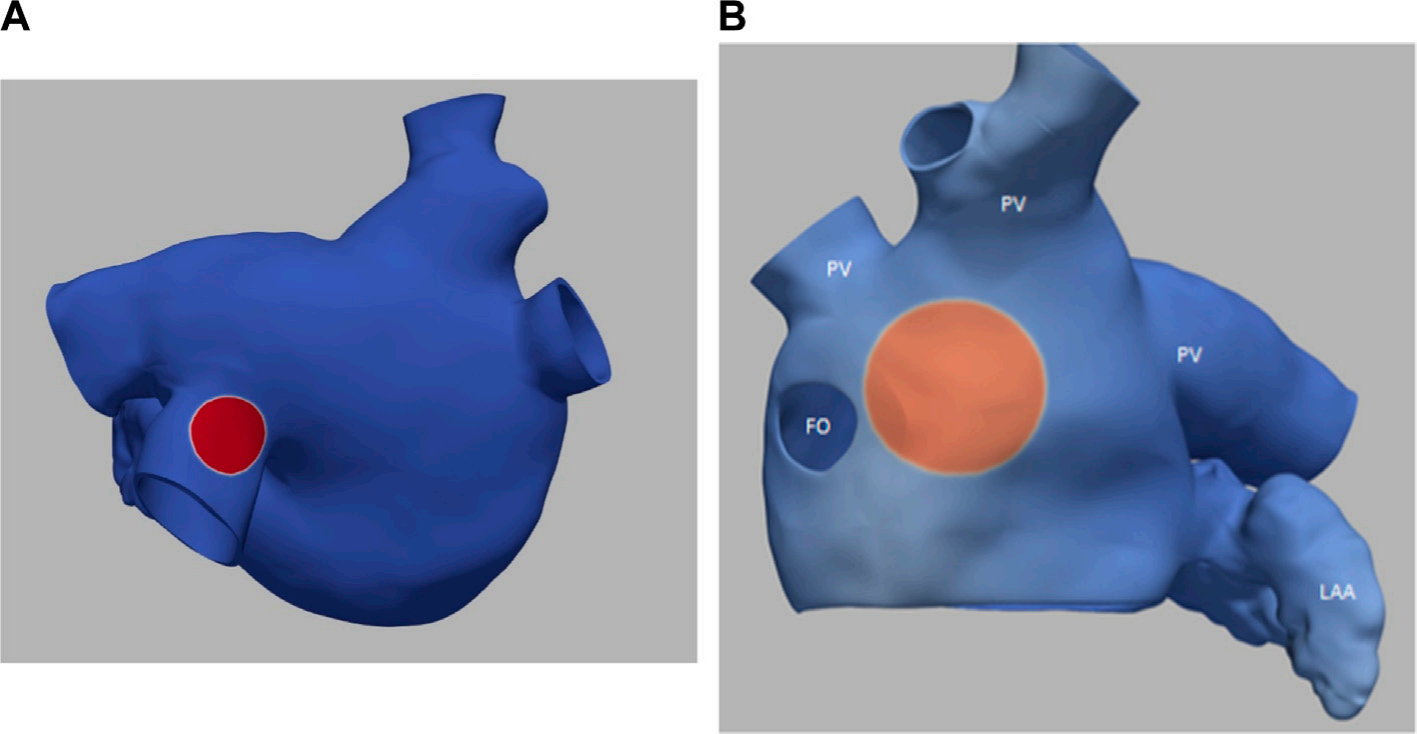
**(A)** small S1 site (radius of 0.5 cm) **(B)** large S2 site (radius of 1.0 cm). Transmembrane potential is represented with a blue-red color map such that blue corresponds to −90 mV and red to +30 mV. The hole on the left in panel B is the fossa ovali (FO)s, the extensions represent pulmonary veins (PV) and the left atrial appendage (LAA) is in the bottom right.

**FIGURE 4:**
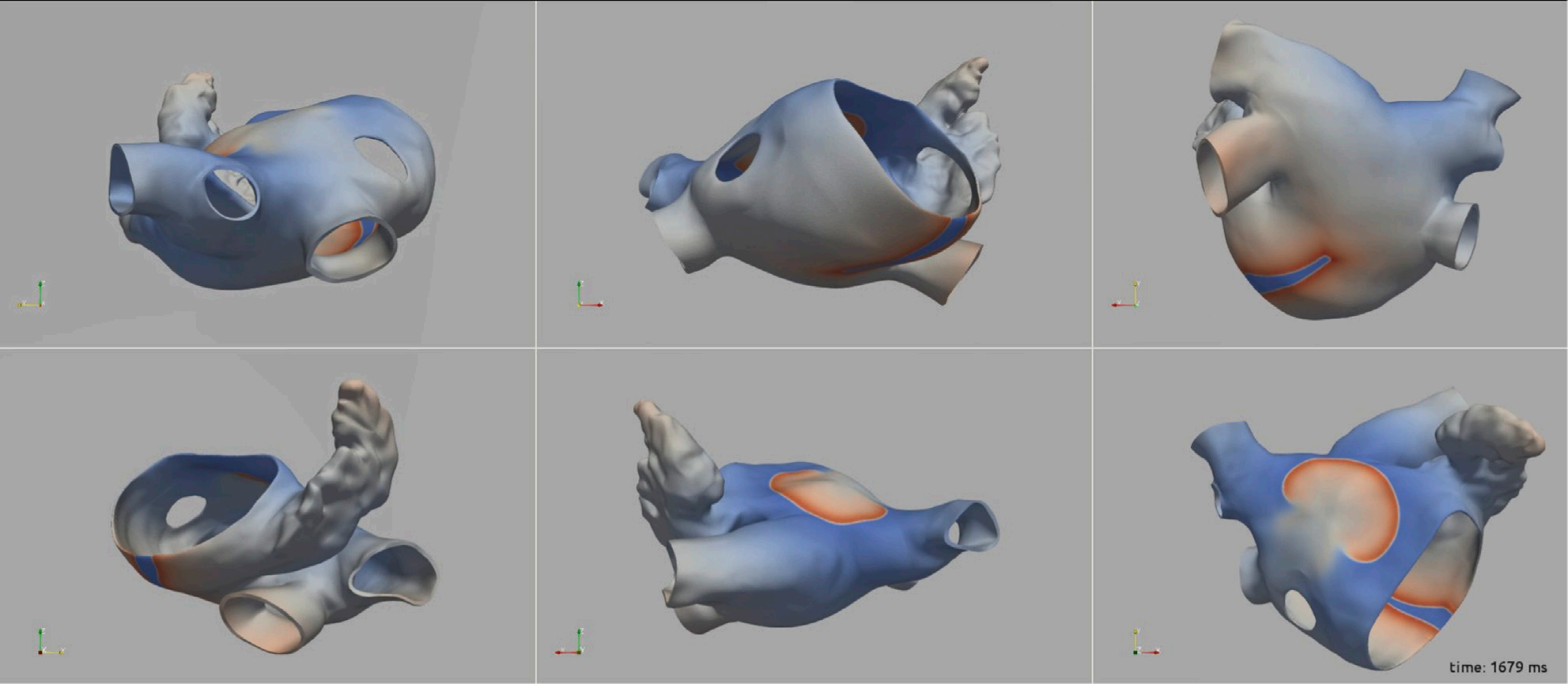
A snap shot of the reentry simulation in multiple view angles at 1,679 ms into simulation. Transmembrane potential is represented with a blue-red color map such that blue corresponds to −90 mV and red to +30 mV.

**FIGURE 5:**
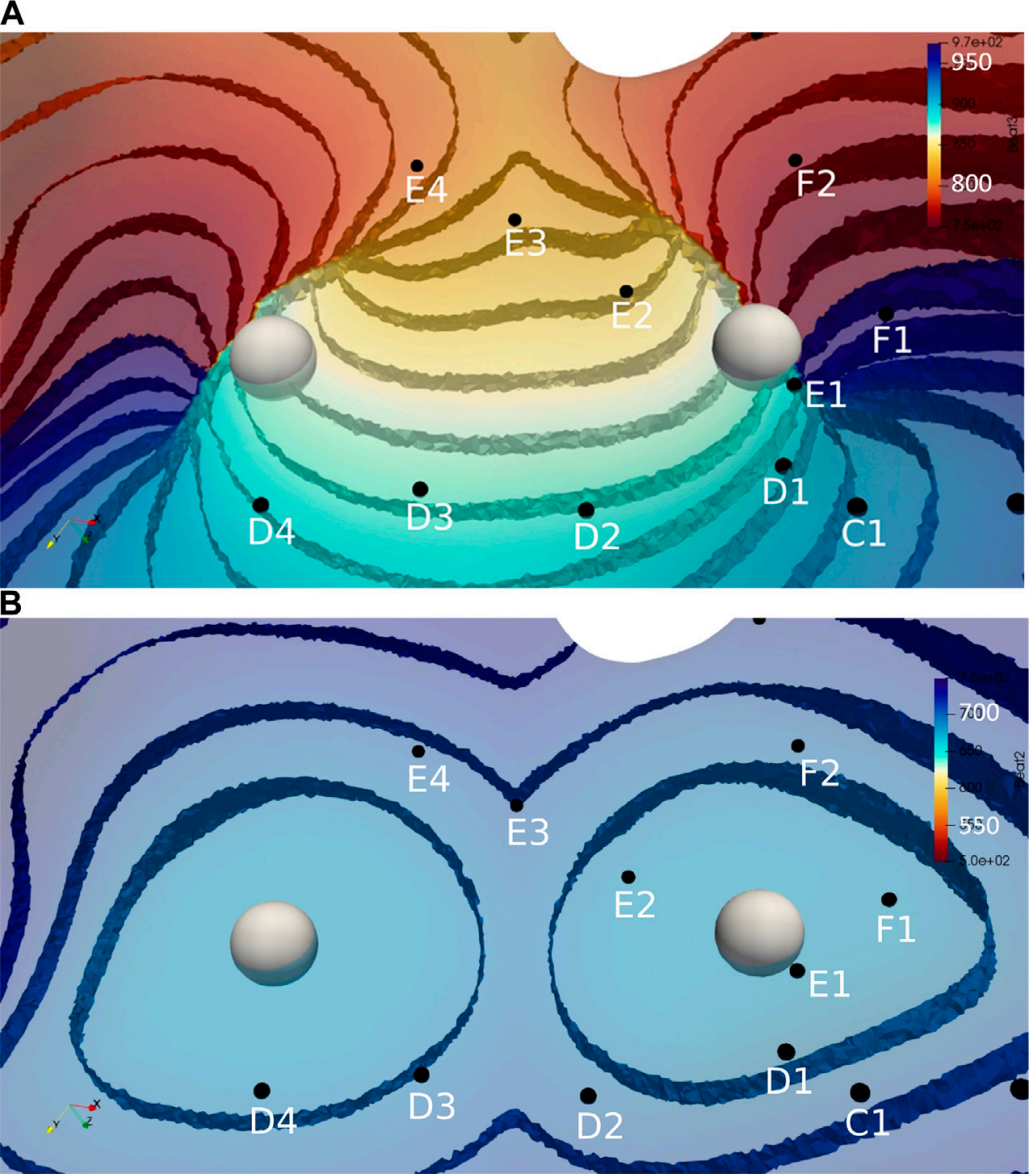
Activation time isosurfaces with points of reentry (spheres) for **(A)** Reentry simulation **(B)** Focal simulation. Surface electrodes are marked by black dots with spline label in white. The color bar represents the activation times for beat 2, red 500 ms and blue 970 ms.

**FIGURE 6:**
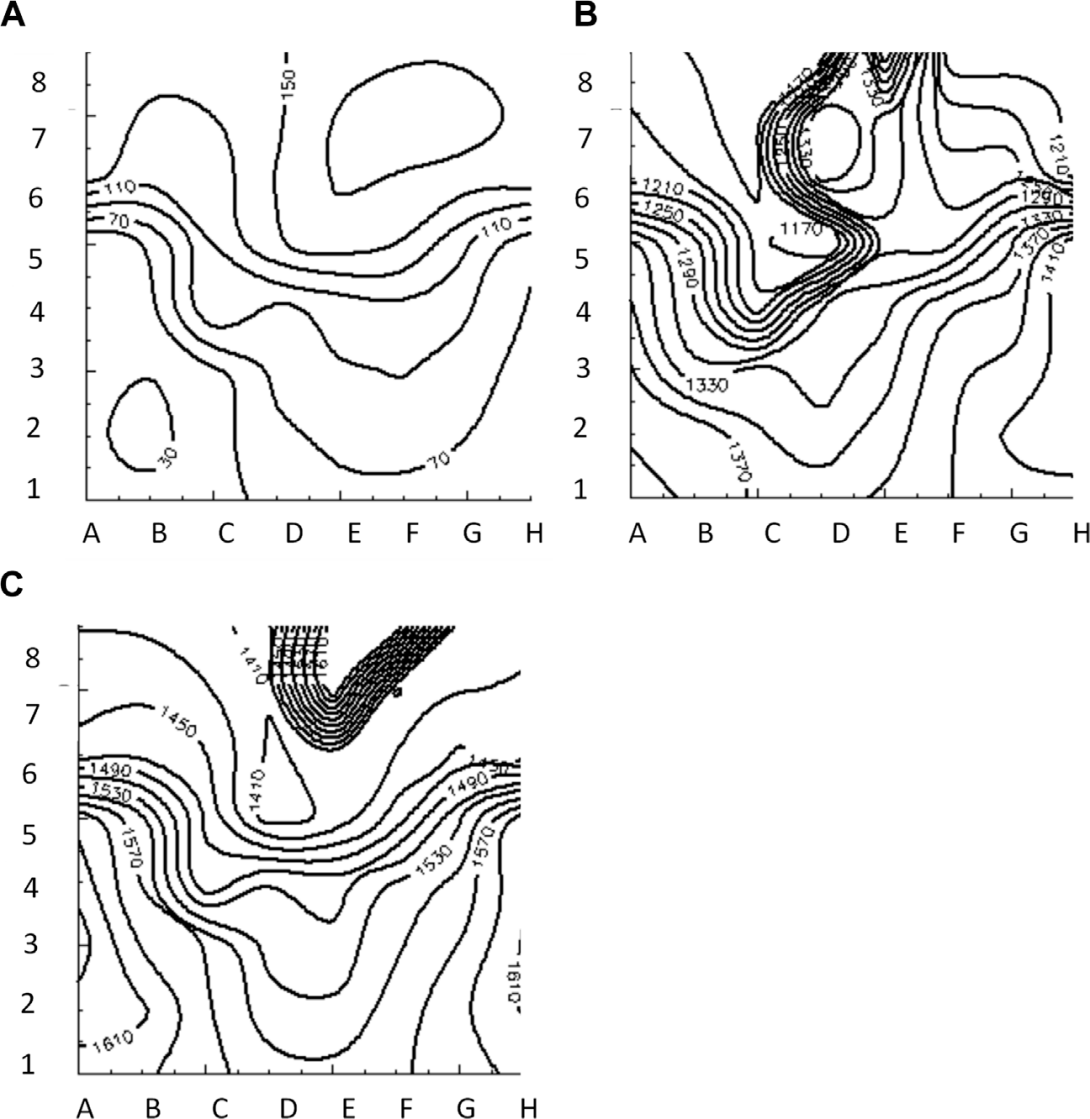
Isochrone maps of paced **(A)**, reentrant **(B)** and focal **(C)** activation patterns computed from 8 × 8 grid of electrodes for BGC.

**FIGURE 7:**
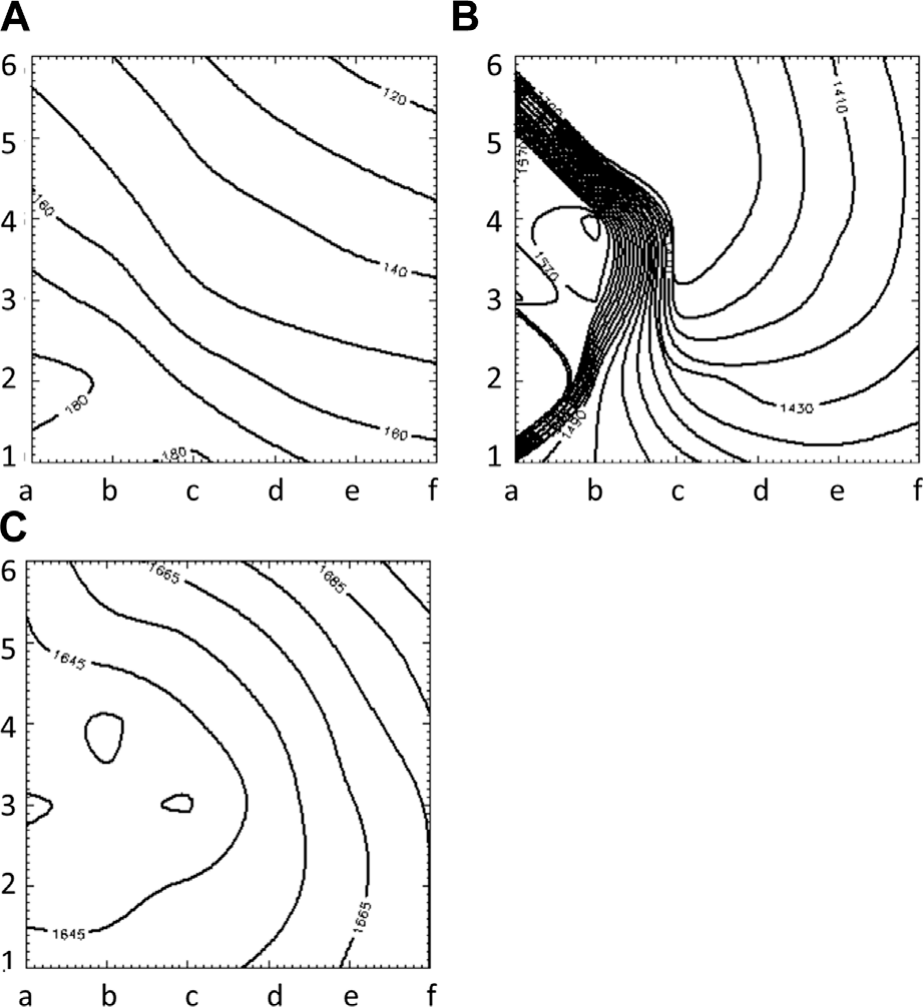
Isochrone maps of paced **(A)**, reentrant **(B)** and focal **(C)** activation patterns computed from 6 × 6 grid of electrodes for UA.

Figure 3 illustrates the initiation of the paced beat (Panel A) and the location of the S2 stimulus which was applied 390 m after the paced stimuli (Panel B). A snapshot of activity from 6 views is shown in Figure 4 illustrating the figure-of-eight reentrant pattern. A video of the simulation is provided as a Supplementary Material. The figure-of-eight reentrant patterns from this simulation and focal patterns from the second simulation on the endocardial surface are shown in Figure 5 with the computed centers of mass of R+ and R-displayed as grey spheres. The location of these patterns in relation to the LA can be ascertained by viewing Figure 2A and the isochrone maps constructed from the 8 × 8 grid of electrodes for BGC for paced, reentry and focal activity in Figure 6. Similarly, isochrone maps constructed from the 6 × 6 grid of electrodes for UA are shown in Figure 7.

### 3.2 Comparison of mapping system output and simulation results

**FIGURE 8:**
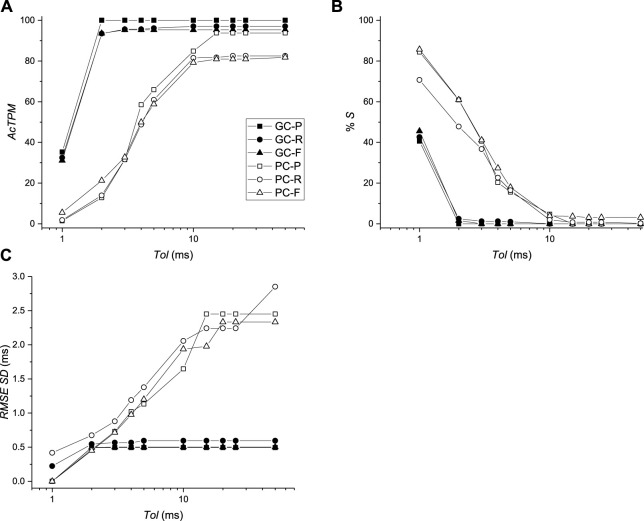
Comparison of activation times between virtual mapping systems BGC and BPC and simulations. **(A)** Activation time performance metric (*AcTPM*) defined in Eq. 5 as a function of tolerance (*Tol*). **(B)** Percentage of spurious AcTs as a function of *Tol*. **(C)** RMS standard deviation (SD) as a function of *Tol*.

The average RMS value of the difference of AcTs computed from $V_{m}$ and $\emptyset_{e}$ signals was less than 1 ms for all beats for all values of *Tol* ranging from 0 to 100 ms for both BGC and BPC. In fact, all values were below 0.24 ms except for the paced beat for BPC, for which the average RMS was between 0.38 and 0.58 ms. The fact that all values were less than 1 ms motivated the development of the novel performance metrics presented in the Methods Section. *AcTPM* and %*S* values are shown for BGC and BPC as a function of *Tol* for P, R, and F patterns in Figure 8. As expected *AcTPM* was always larger, and % S was always smaller, for BGC compared to BPC for all activation patterns. The trend was for *AcTPM* values to increase and % *S* values to decrease as *Tol* increased and reach plateau values with these values being less for BPC compared to BGC, and highest for P as compared to R and F for both BGC and BPC. The plateau for BGC was reached at *Tol* = 2 ms, where the corresponding value for BPC was *Tol* ≈ 10 ms. For BGC, the values for *AcTPM* for *Tol* = 2 ms were 100, 93.5 and 93.6 for P, R, and F respectively and the corresponding values for %*S* were 0.2, 2.5, and 1.8. For BPC, the values for *AcTPM* for *Tol* = 10 m were 84.9, 79.2 and 81.5 for P, R, and F respectively and the corresponding values for % *S* were 4.7, 2.0, and 3.9. Although the average RMS values were always less than 1 ms, the RMS SD was a function of Tol and was much greater for BPC compared to GC (Figure 8C).

The ability of the MSs (BGC, BPC, and UA) to identify the one paced (P) beat, the twelve reentrant (6 R+; 6 R-) patterns, and the 12 focal patterns (6 F+; 6 F-) are presented in Table 2. This comparison was carried out for two values of δ (2 and 10 ms) which corresponds to the values for which *Tol* reached plateau values for BGC and BPC respectively. The P beat was not identified for any MS (hence AcPPM = 0) with one misclassification and one spurious patterns evident for BGC only. Only 1 of 6 R-beats were identified for BGC (with 0 and 5 spurious patterns for δ = 2 and 10 m, respectively). Four (*δ* = 2 ms) or five (*δ* = 10 ms) of 6 R+ beats were identified by BGC and 2 of 6 for BPC (*δ* = 10 ms) while 5/6 were identified for UA; UA resulted in no spurious patterns while there were 0 for both BGC and BPC (*δ* = 2 and 10 m); the only misclassifications of R+ occurred for BGC, δ = 10 ms. Focal beats were identified with temporal error less than 10 ms for BGC, BPC, and UA, although only F+ beats were identified for UA (which is consistent with its placement, see Figure 2C). Overall, F beats were easier to identify than R beats for our simplified algorithm.

**TABLE 2:** Value of ACPPM of Paced (P), Reentrant (R), and Focal (F) patterns, with temporal (*E*_*T*_) and spatial (*E*_*X*_) errors as a function of *δ*; when ACPPM = 0, the number of spurious (S) and misclassifications (M) are presented.

*AcPPM* (%)	P: (*E*_*X*_ cm; *E*_*T*_ ms)	R+: (*E*_*X*_ cm)	R-: (*E*_*X*_ cm)	F+: (*E*_*X*_ cm; *E*_*T*_ ms)	F-: (*E*_*X*_ cm; *E*_*T*_ ms)
BGC (*δ* = 2)	0 1S, 1M	67 (0.52)	17 (0.63)	83 (0.52; 0.0)	50 (0.63; 5.7)
BPC (*δ* = 2)	0 0S, 0M	0 0S, 0M	0 0S, 0M	83 (0.5; 0.0)	0 0S, 0M
BGC (*δ* = 10)	0 1S, 0M	69 (0.47)	2.3 (0.63)	69 (0.52; 0.0)	83 (0.68; 7.3)
BPC (*δ* = 10)	0 0S, 0M	33 (0.41)	0 0S, 0M	69 (0.57; 1.7)	83 (0.83; 9.4)
UA (*δ* = 2)	NA 0S, 0M	83 (0.34)	NA 0S, 0M	83 (0.37; 0.0)	0 (NA)
UA (*δ* = 10)	NA 0S, 0M	83 (0.37)	NA 0S, 0M	69 (0.47; 2.2)	0 (NA)

Two factors that affect the ability of a MS to identify patterns on the heart surface are: 1) the distance of the electrodes from the heart surface; and 2) the density of the surface projection of the MS electrode sites. These two values for each electrode are shown in Table 3 for BPC; the first number is the distance of the electrode to the nearest endocardial mesh node, and the second number is the average distance to the eight nearest projected endocardial sites. The fact that the location for nearest electrode for R+, F+ (E2) was closer and had a higher surface density compared to the location of the nearest electrode for R-, F- (D4) is consistent with the trend of better identification of + patterns sites compared to—patterns. To demonstrate the effect of these factors, the pattern identification algorithm described above was applied to the AcTs computed from the noiseless $V_{m}$ signals. These values of *AcPPM* for $V_{m}$ data were: P: 0 (1S); R+: 69; R-:14; F+: 83; F-:100 for δ = 2 m. These values are similar to BGC (*δ* = 2 ms) suggesting that BGC performed nearly as well as could be expected (except for F- suggesting that the optimal electrode resolution might fall between E2 and D4, see Table 3). The actual *x,y,z* location of R+ and F+ beats was located 0.52 cm from the nearest surface site which corresponded to B7. The fact that electrode B7 corresponds to a high surface projection density and a small electrode to surface distance (0.18 cm) explains why F+ was the only activation pattern identified by BPC (*δ* = 2 ms).

**TABLE 3:** Distance between electrodes for BPC and endocardial surface (in cm), average distance of 8 nearest neighbors of 64 projected endocardial sites (in cm). Electrodes near the poles of the sphere do not have 8 neighbors so the second value is undefined (NA).

	A	B	C	D	E	F	G	H
1	0.12, NA	0.06, NA	0.04, NA	0.09, NA	0.16, NA	0.19, NA	0.21, NA	0.19, NA
2	0.29, 0.65	0.14, 0.65	0.13, 0.74	0.17, 0.85	0.18, 0.88^a^	0.21, 0.86	0.32, 0.91	0.37, 0.77
3	0.50, 1.48	0.30, 1.17	0.30, 1.14	0.27, 1.10	0.23, 1.10	0.26, 1.12	0.42, 1.4	0.62, 1.76
4	0.63, 1.89	0.37, 1.57	0.46, 1.60	0.43, 1.61^b^	0.37, 1.64	0.43, 1.58	0.52, 1.73	0.68, 2.14
5	0.42, 1.27	0.30, 1.43	0.62, 2.10	0.57, 1.80	0.47, 1.61	0.33, 1.40	0.35, 1.20	0.43, 1.31
6	0.24, 1.05	0.39, 1.56	0.60, 1.61	0.39, 1.17	0.28, 0.99	0.19, 0.99	0.18, 0.95	0.23, 1.01
7	0.14,0.82	0.30, 0.95 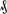	0.38, 0.96	0.26, 0.81	0.16, 0.79	0.09, 0.77	0.07, 0.77	0.10, 0.77
8	0.1, NA	0.18, NA	0.21, NA	0.17, NA	0.11, NA	0.05, NA	0.04, NA	0.05, NA

^a^ Surface electrode nearest to R+ and F+ location.

^b^ Surface electrode nearest to R- and F- location.

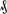
surface electrode nearest to P location.

## 4 Discussion

The success of an electrophysiological procedure to localize and ablate arrhythmogenic sources in the heart depends on a variety of interrelated factors such as: the patient’s heart geometry, disease state, and arrhythmia; the number, type, and location of recording electrodes; mapping system hardware and software (algorithms); data display; and physician interpretation. These can be summarized into four distinct categories as shown in Figure 1: 1) the heart; 2) the mapping system; 3) the display; and 4) the physician.

In this manuscript we present a novel computational framework that enables a rigorous evaluation of a mapping systems ability to localize the arrhythmogenic sources and their type (i.e., focal or reentrant), which spans categories 1) and 2), via a blinded comparison with numerical simulations. As far as we are aware, the only other similar study focused on a computational framework for MS evaluation was by Bartolucci et al., 2021 in which the results from two virtual catheters were compared using simulations of a two-dimensional spiral wave (Bartolucci et al., 2021). Here, activation times and patterns for virtual $\emptyset_{e}$ signals were computed for simulations incorporating three hypothetical MSs (BGC, BPC, and UA) and compared to the corresponding high resolution $V_{m}$ data from two simulations containing paced (P), reentrant (R), and focal (F) patterns. We introduce two novel ‘quantitative performance metrics’ (QPMs); one for patterns (AcPPM) and one for activation times (AcTPM) because RMS error was not indicative of performance. These QPMs reflect the ability of the MS to identify AcTs and AcPs, respectively by combining the number of correctly identified, spurious, and misclassified AcTs and AcPs. Identifying “correct” AcTs and AcPss from electrogram data requires choosing “error tolerances” for continuous variables and these choices most likely will impact the QPMs. Therefore, we believe it is important to be transparent and clear regarding these error tolerance choices. The choices in this work are the threshold derivative for virtual $\emptyset_{e}$ AcTs (*a*); AcT similarity tolerances (*Tol* and δ); an interval threshold for neighboring AcT to ensure they are part of the same wavefront (*γ*); as well as distance (1 cm) and time (100 ms) thresholds when comparing patterns to the simulations. Each of these “tolerance parameters” (TPs) will affect the performance evaluation (see Table 3); therefore we suggest identifying the sensitivity of the QPMs to these TP values. In addition, these TPs could be constrained based on important clinical factors (e.g., ablation lesion size). We believe that much further work is required to identify the best QPMs and TPs for a computational framework for clinical MS evaluation. Regardless of the choices, we believe that the ideal computation of QPMs should include an analyses of the sensitivity to TPs and relevant simulation parameters (e.g., noise) as well as the consideration of uncertainty (including measurement uncertainty).

It is well understood that the specific activation patterns in the heart are dependent on the underlying mechanism of the patient’s arrhythmia and that the corresponding sources can be either focal or reentrant, which can be difficult to distinguish with a limited number of electrogram recordings (Li et al., 2020). In addition, comparison studies involving retrospective analyses of clinical data have shown both similar (Alhusseini et al., 2017; Podziemski et al., 2018; Swerdlow et al., 2019) and disparate (Luther et al., 2017; Anter et al., 2018) results regarding mapping system algorithms. A computational approach to MS evaluation will aid in not only making these issues transparent but also in providing a framework to quantify these effects. The fact that BGC and simple algorithms performed poorly in identifying AcPs (see Table 2) was the result of inadequate sampling capture patterns as demonstrated by similar results when analyzing the nearest 64 transmembrane signals.

As expected, we found that the following two issues were the primary factors contributing to the ability of a mapping system to correctly identify activation patterns: 1) the distance from the electrodes to the heart surface; 2) the physical location of each activation pattern in relation to the density of the projection of the electrodes onto the heart surface. This finding is consistent with previous studies. For example, Alessandrini et al. computed extracellular electrograms during simulated AF in a patient-specific LA using models of grid catheters as well as realistically deformed basket catheters (Alessandrini et al., 2018). They found that computed maps of rotor tip trajectory density correctly identified and located the virtual rotors (deviation <10 mm) only for catheter recordings of sufficient resolution (inter-electrode distance ≤3 mm) and proximity to the wall (≤10 mm). In addition, Roney et al., performed simulations to estimate the minimum number of measurement points required to correctly identify the underlying AF mechanism and found that the spatial resolution required for correct identification of rotors and focal sources was a linear function of spatial wavelength (the distance between wave fronts) of the arrhythmia (Roney et al., 2017a). They also found that all clinical high-resolution multipolar catheters are of sufficient resolution to accurately detect and track rotors when placed over the rotor core, although the low-resolution basket catheter was prone to spurious detections and may incorrectly identify rotors that are not present (Roney et al., 2017a). Martinez-Mateu et al. (Martinez-Mateu et al., 2019) also identified two different types of ‘phantom rotors’ associated with basket catheters due to the far-field sources and to the interpolation between the electrodes and found that the ability to detect rotors depended on the basket’s position and the distance between the electrodes and the heart surface.

The goal in this study was to develop a framework for evaluating MSs, therefore the choice of QPMs are most likely not be optimal, in part due to a variety of limitations. First, the specific comparison analyses presented here depend on: 1) the electrode configuration; 2) the simple MS algorithms we employed; 3) the noise level as well as its spatial uniformity and 4) the specific type and location of electrical patterns in the simulations as well as the choice of cell model (e.g., including “remodeling” may be appropriate for simulating AF (Heijman et al., 2021)). For this pilot study the RMS error of AcTs was less than 1 ms indicating that activation pattern reconstruction of electrograms will be similar to those computed from “down sampling” the transmembrane action potentials from the endocardial surface. AcTs may not correspond well to action potential depolarization during situations in which propagation is abnormal (e.g., at sites of fractionization during persistent AF). More sophisticated MS algorithms than those used here that include spatial and temporal interpolation might improve the identification of AcPs, although care must be taken to interpolate phase values correctly (Roney et al., 2017a; Jacquemet, 2018). Incidentally, our simple MS algorithms did not include any phase calculations; preliminary tests to identify patterns using phase showed decreased performance in identifying reentry compared to the algorithms presented here based on AcTs only. Second, the simulations presented here were carried out using an isotropic left atrium (only) derived from a healthy male. A computational study by Jacquemet et al. (Jacquemet et al., 2003) provides insight into the impact of atria structure on electrogram morphology: they concluded that regardless of anisotropy, wavefront collisions are not the basis of multiphasic electrograms during AF. Third, we implemented a specific “basket-like” geometry which does not capture certain aspects of the clinical situation (Laughner et al., 2016; Oesterlein et al., 2016; Honarbakhsh et al., 2017). Fourth, this study ignored electrogram morphology and only considered the time of activations (i.e., only AcTs were computed using a simple threshold of maximum derivative); in order to support the practical usefulness of this framework to incorporate electrogram morphology validation of virtual electrode signals with clinical signals would be required. Nevertheless, our study includes a quantitative comparison of three hypothetical electrode configurations with the same reference standard (i.e., simulation results) and the same MS algorithms.

A very important question is “How well do the Quantitative Performance Metrics (QPMs) of a MS, resulting from challenging the MS with simulated electrograms from computer simulations, predict real-world performance of the same MS in the intended use population?” Ideally, this would be addressed by performing validation of the MSEF Framework. This would involve comparing conclusions from MSEF MS evaluation with conclusions from clinical MS evaluation. However, we expect this approach to be very difficult and possibly unethical.

FDA is responsible for ensuring the reasonable assurance of safety and effectiveness of medical products in the United States using the following definition of effectiveness defined in Section 860.7(e) (1) of the Code of Federal Regulations: “There is reasonable assurance that a device is effective when it can be determined, based upon valid scientific evidence, that in a significant portion of the target population, the use of the device for its intended uses and conditions of use, when accompanied by adequate directions for use and warnings against unsafe use, will provide clinically significant results.” We believe that this study represents a major step in establishing appropriate performance criteria for MSs using a computational simulation framework. However, discussions with the MS and clinical community regarding appropriateness, justification, and validation will help further refine the framework and next steps in development of MSEF tools such as generating an appropriate library of computational simulations; identifying and standardizing appropriate performance metrics; validating the approach; and automating the steps identified in Table 1.

Our MSEF is based on well-established scientific methods and provides results in the form of two new performance metrics. We believe that our MSEF provides significant new information to aid in the performance evaluation of cardiac mapping systems which is necessary to assess effectiveness. The results can be used to identify the performance of a specific mapping system as a function of a variety of variables, and due to the use of computer simulations the framework is flexible to account for a multitude of inter-related factors that depend of the context of use of the system. Identifying the number and type of simulations to include in the library is extremely challenging; ideally they would represent the geometry, patterns, and electrogram morphology representative of the patient population of interest. Incidentally, the framework can incorporate simulation results from a combination of super computers, graphical processing units (Kaboudian et al., 2010), or desktop computers (Pathmanathan and Gray, 2015; Galappaththige et al., 2019), depending on the level of desired fidelity. Overall, our results demonstrate the feasibility of a computational framework as a method for quantitatively evaluating the performance of mapping system algorithms that compute activation time and/or analyze activation patterns.

## Data Availability

The raw data supporting the conclusion of this article will be made available by the authors, without undue reservation.

## Supplementary material

SUPPLEMENTARY FIGURE S1 Effect of noise on the comparison of activation times between virtual mapping systems BGC and BPC and simulations (black: no noise; red: noisex2; green: noisex3). **(A)** Activation time performance metric (*AcTPM*) defined as a function of tolerance (*Tol*). **(B)** Percentage of spurious AcTs as a function of *Tol*. **(C)** RMS standard deviation (SD) as a function of *Tol*.

SUPPLEMENTARY VIDEO S1 AF_test14.mpg https://www.researchgate.net/publication/366893522_AF_Test14.

SUPPLEMENTARY VIDEO S2 Focal_AF_test14.mpg https://www.researchgate.net/publication/366893430_Focal_AF_Test14.

## Abbreviations

AcP, Activation Pattern; AcPPM, Activation Pattern Performance Metric; AcT, Activation Time; AcTPM, Activation Time Performance Metric; AF, Atrial Fibrillation; BGC, Basket Good Contact virtual electrode catheter; BPC, Basket Poor Contact virtual electrode catheter; CAD, Computer Aided Design; E_T_, temporal localization error; E_X_, spatial localization error; F, F+, F-, Focal beat, + = at location of R+, - = at location of R-; *fC*, Fraction Correct; *fM*, Fraction Misclassification; *fS*, Fraction Spurious; LA, Left Atria; MS, Mapping System; MSEF, Mapping System Evaluation Framework; P, Paced beat; PS, Phase Singularity; QPM, Quantitative Performance Metric; R, R+, R-, Reentrant beat, + = clockwise, - = counter-clockwise; *Tol*, Tolerance; UA, Uniform Array virtual electrode catheter; VF, Ventricular Fibrillation; *V*_*m*_, transmembrane potential.
