# Supplementary material for: A computational modeling framework for pre-clinical evaluation of cardiac mapping systems
Source: Front Physiol. 2023 Jul 6;14:1074527. doi: 10.3389/fphys.2023.1074527 (PMC10358980; doi:10.3389/fphys.2023.1074527)
Supplement: Supplementary file 1 [file DataSheet1.pdf]

Supplementary Material

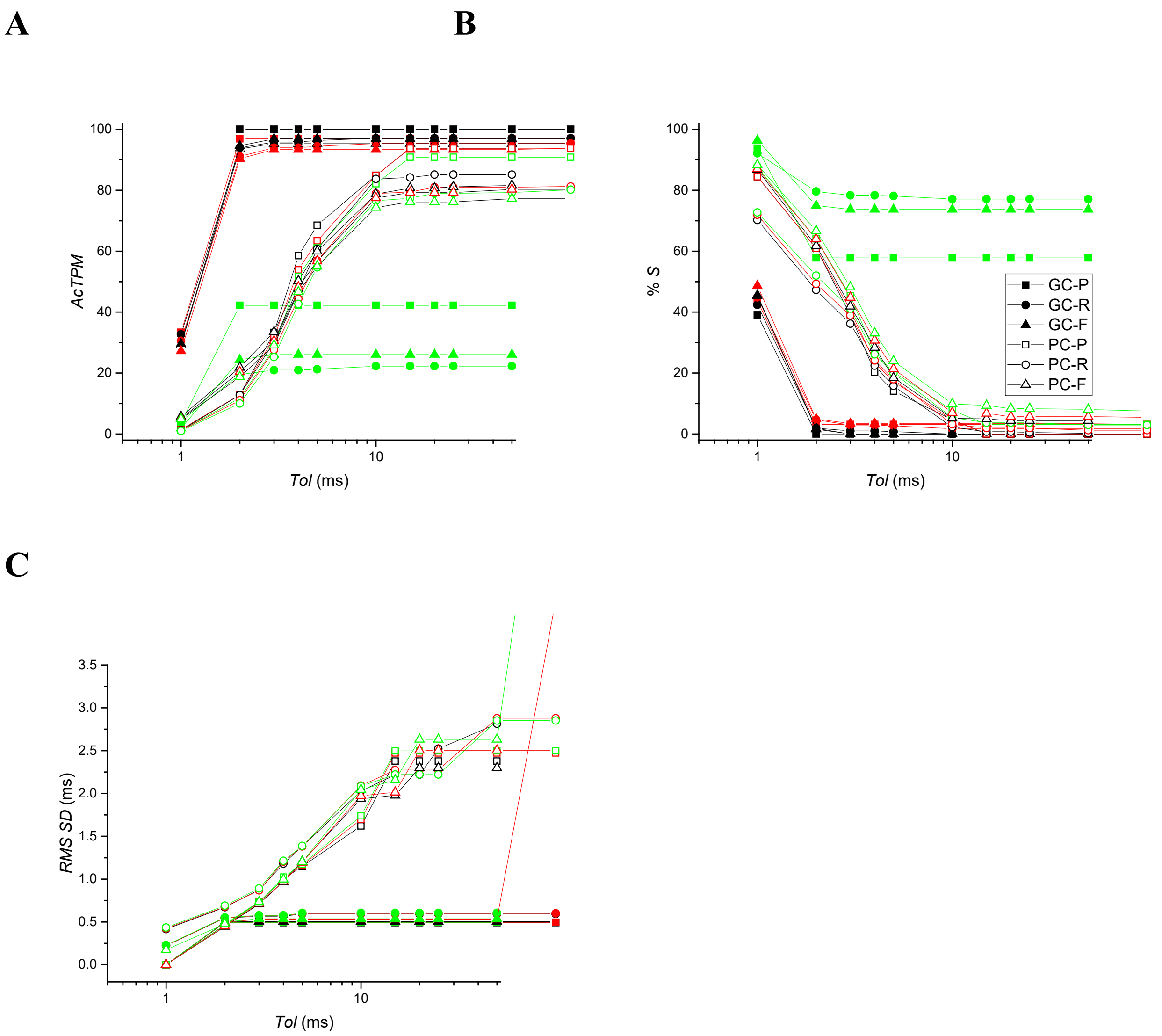

Figure S1. Effect of noise on the comparison of activation times between virtual mapping systems BGC and BPC and simulations (black: no noise; red: noisex2; green: noisex3). A) Activation time performance metric (*AcTPM*) defined as a function of tolerance (*Tol*). B) Percentage of spurious AcTs as a function of *Tol*. C) RMS standard deviation (SD) as a function of *Tol*.

Video Files:

AF_test14.mpg

https://www.researchgate.net/publication/366893522_AF_Test14

Focal_AF_test14.mpg

https://www.researchgate.net/publication/366893430_Focal_AF_Test14
